# AI-driven Eyeball Exposure Rate (EER) analysis: A useful tool for assessing ptosis surgery effectiveness

**DOI:** 10.1371/journal.pone.0319577

**Published:** 2025-03-25

**Authors:** Byungchul Lee, Lianji Xu, Sang-Ha Oh, Yooseok Ha, Hyeokjae Kwon, Kyu Cheol Lee, Soo Yeon Kim, Chang Wook Seo, Sunje Kim, Seung Han Song

**Affiliations:** 1 Department of Plastic and Reconstructive Surgery, Chungnam National University Hospital, Daejeon, South Korea; 2 Department of Plastic and Reconstructive Surgery, Beijing Tongren Hospital, Capital Medical University, Beijing, China; 3 Department of Medical Science, College of Medicine, Chungnam National University, Daejeon, Republic of Korea; 4 Department of Plastic and Reconstructive Surgery, College of Medicine, Chungnam National University, Daejeon, Republic of Korea; 5 CheongdamRui Plastic Surgery Hospital, Jeju, South Korea; 6 Kimbelle Dermatology Hospital, Daejeon, South Korea; 7 Anigma Technologies Incorporated, Daejeon, South Korea; Chitkara University Institute of Engineering and Technology, INDIA

## Abstract

**Introduction:**

Ptosis surgery outcomes are measured by one-dimensional metrics like Marginal Reflex Distance (MRD) and Palpebral Fissure Height (PFH) using ImageJ. However, these methods are insufficient to capture the full range of changes post-surgery. Eyeball Exposure Rate (EER) offers a more comprehensive two-dimensional perspective as metric. This study compares AI-based EER measurements with conventional ImageJ methods for assessing outcome of ptosis surgery. Methods: Images from 50 patients (total 100 eyes) taken before and after surgery were analyzed using manual ImageJ and the AI-tool “Anigma-View”. Statistical tests assessed the accuracy and consistency of both methods, using intraclass correlation coefficients (ICCs) and Bland-Altman plots for comparison.

**Results:**

EER measured by the AI-tool at pre- and post-operation were 58.85% and 75.36%, respectively. Similarly, manual measurements using ImageJ showed an increase from 58.22% to 75.27%. The Intraclass Correlation Coefficients (ICCs) between the AI-tool and manual measurements ranged from 0.984 to 0.994, indicating excellent agreement, with the repeated AI-tool demonstrating high reproducibility (ICC =  1). Bland-Altman plots showed excellent agreement between the two methods and reproducibility of AI-based measurements. Additionally, EER improvement was more prominent in the moderate to severe ptosis group with a 45.94% increase, compared to the mild group with 14.39% increase.

**Discussion:**

The findings revealed no significant differences between AI-tool and manual methods, suggesting that AI-tool is just as reliable. AI-tool to automate measurements offers efficiency and objectivity, making it a valuable method in clinical fields.

**Conclusion:**

AI-based EER analysis is accurate and efficient, providing comparable results to manual methods. Its ability to simplify surgical outcome assessments makes it a promising addition to clinical practice. Further exploration of AI in evaluating three-dimensional changes in ptosis surgery could enhance future surgical assessments and outcomes.

## Introduction

Ptosis, also known as drooping of the upper eyelid, is a frequent problem observed among Koreans, and ptosis surgery is one of the most common procedures in plastic surgery fields. Analyzing the outcomes of ptosis surgery often relies on retrospective image analysis, comparing the pre- and post-surgical results to evaluate the surgical outcomes. General methods for evaluating outcomes of ptosis surgery depend on several one-dimensional metrics, including Marginal Reflex Distance (MRD) and Palpebral Fissure Height (PFH) [1,2].

MRD1 is the distance from the center of the pupil to the upper eyelid margin, which is a well-recognized metric for assessing upper eyelid position. Similarly, PFH assesses the vertical distance between the upper and lower eyelid margins at the pupil’s center. Nevertheless, these measurements are one-dimensional and may not fully reflect the complex, three-dimensional changes in the eye's appearance after surgery [3].

**Fig 1:**
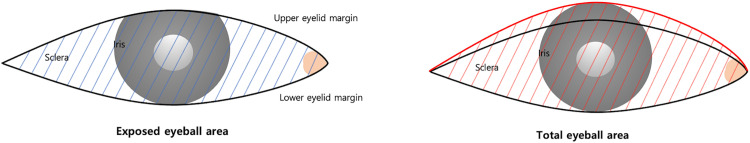
Schematic drawing of eyelid parameters.

The Eyeball Exposure Rate (EER) is a more comprehensive, two-dimensional metric that reflects the exposed areas of the sclera and iris. EER is calculated by measuring the ratio of the exposed corneal and scleral areas between the upper and lower eyelids [4]. This metric can reflect the overall appearance and changes of eye exposure in two-dimensions (Fig 1).

ImageJ, a widely used software in plastic surgery research, is the conventional tool for measuring both lengths and areas in medical images. The analysis values of photographs using ImageJ are considered reliable and being used in many studies [5]. It is known as a manual approach, in which the evaluator has to physically mark and measure each image, and its result depends on the expertise of the evaluator [6,7].

In recent years, AI-based technologies have been developed for the process of image analysis in medical research [6]. These AI-tools can measure images automatically, resulting in faster, more reliable measurements [6–9].

Despite the increasing adoption of AI technologies, direct comparisons of the accuracy and reproducibility of AI-based EER measurements with those obtained using ImageJ remain scarce. This study aims to address this limitation by assessing the accuracy, reproducibility, and clinical applicability of AI-based tools for measuring EER before and after ptosis surgery, providing insights into their potential as reliable methods.

## Methods

### Study participants and ethics statement

This study examined 100 eyes from 50 patients, all aged over 19 years, who visited Chungnam National University Hospital (CNUH) in the CNUH Department of Plastic and Reconstructive Surgery between July 24, 2023 and July 5, 2024. The study included 50 patients with bilateral ptosis who underwent blepharoptosis surgery. All surgeries were performed by a single expert with over 20 years of experience. These 50 patients group consisted of 25 men and 25 women, ranging in age from 19 to 80 years. Of these patients, 33 had equal ptosis severity in both eyes, while 17 had varying degrees of severity between their eyes. The study, including a total 100 eyes, classified 51 eyes as having mild ptosis and 49 as moderate to severe. Ptosis severity was classified based on the extent of upper eyelid drooping: mild ptosis was defined as drooping of 1–2 mm, while moderate to severe ptosis involved drooping of more than 3 mm [10]. The study excluded patients with a history of previous eye surgeries or facial trauma. Demographic information, such as age and sex, was collected and analyzed (Table 1). The study conducted a retrospective analysis of photographs taken pre-operation and six months post-operation.

**Table 1:** Patient demographics and severity.

Demographics	
**No. of patients (no. of eyelids)**	50 (100)
**Age (yr)**	46.9 ± 18.7*
**Sex (male/female)**	25/25
**Severity of ptosis (no. of eyelids)**	
**Mild ( < 2 mm)**	51
**Moderate–Severe ( **≥ **2 mm)**	49

*Values are presented as mean ±  standard deviation.

This study was approved by Ethics Committee of Chungnam National University Hospital, Daejeon, Korea (CNUH-IRB No. 2023-06-098) and performed following the ethical guidelines of the Declaration of Helsinki. All data were anonymized and data were accessed for research purposes from July 24, 2023 to July 19, 2024. Informed written consent was obtained from all participants, including consent for potential publication in both online and print formats.

### Image acquisition

Photographs of all patients were taken by the same photographer from a frontal view at two time points: before surgery and six months after surgery. For all photo sessions, patients were seated about 1.0 m away from the camera, maintaining a natural head position (NHP) aligned along the same horizontal axis. When images were taken, the patients’ faces and frontalis muscles were relaxed using a digital camera with a standardized lens (Canon 1500 D, Canon Corporation, Tokyo, Japan, 24–120 mm).

### Manual measurements

**Fig 2:**
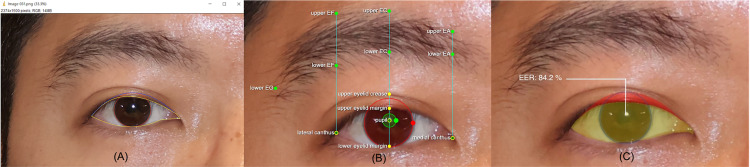
Eyelid measurements using AI-tool and Manual (ImageJ) tool. (A) EER measurement using manual tool (ImageJ 1.46), (B) Eyelid parameters generated by the AI-tool (Anigma-View 1.0.6), (C) EER measurement using the AI-tool (Anigma-View 1.0.6).

All images taken pre- and post-operation were analyzed through ImageJ. The same expert manually measured the EER in the eye area of the image using Java-based software ImageJ version 1.46 (National Institutes of Health, Bethesda, MD, USA) (Fig 2A). The software converted the pixel values to millimeters, using the cornea's inter-limbal distance, standardized at 11.5 mm, as the reference point in each image [11].

### AI-tool measurements

The automated image analysis software for comparisons of preoperative and postoperative EER is “Anigma-View” [12]. Anigma-View version 1.0.6 (Anigma Technologies Inc., Daejeon, Republic of Korea) used in this study is AI-driven software designed for automatic periorbital measurements. The Anigma-View used a DeepLabV3 + [13] model with fine-tuning for semantic segmentation of the eye regions. This software calculates measurements based on a coordinate system where the x and y values originate from the top-left corner of the image. The automated image analysis for eyelid morphology included the following steps (Fig 2B, 2C).

#### Step 1: Image alignment and segmentation.

All images were properly positioned and angled for accurate measurements. The alignment of facial features is crucial to maintain consistent measurements across samples. Following the approach of Lou et al. [8], we used the Face Alignment [14] open-source project to localize the eye regions in the input images. Once aligned, each image underwent segmentation to isolate relevant facial features, facilitating precise calculations. To specifically measure the features in the eye, we cropped the eye area image by 512 ×  512 resolution. This step ensures that our subsequent analysis focuses on the relevant areas of the image.

**Fig 3:**
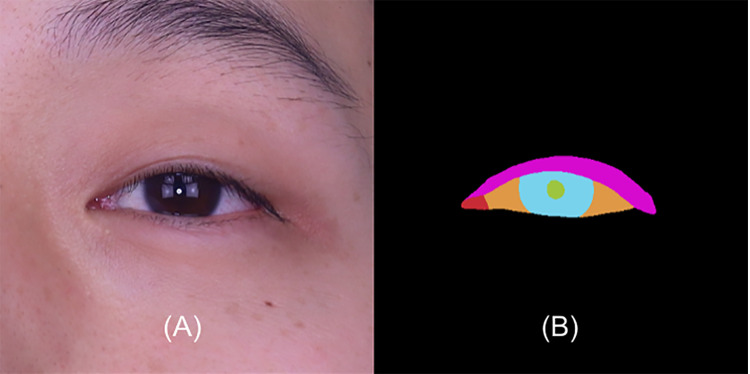
The image is localized to eye regions and segmentation label composes 6 different classes. (A) Input image, (B) Segmentation image.

The localized eye regions were then processed by our fine-tuned DeepLabV3 + model to generate segmentation masks for the eyelid and cornea. To fine-tune DeepLabV3 + [13], we used 396 eye images for training and 50 for testing the model. The segmentation label is separated into 6 classes, background, eyelid, white area, caruncle, iris and pupil. Example data for eye formatting with label pattern is shown in Fig 3.

We fine-tuned DeepLabV3 + with ResNet-101 [15] backbone on our dataset, with input images resized to 512 × 512 pixels. The training process involved 10,000 iterations with a learning rate of 0.0004 using the Adam optimizer. We employed random horizontal flipping and random shifting in a range of -30 to 30 pixels on both x and y axes for data augmentation. The model was trained for 200 epochs with a batch size of 16 using cross-entropy loss. Training was performed on an NVIDIA RTX 3090 GPU using the PyTorch framework. For model evaluation, we employed the mIoU (mean Intersection over Union) metric, achieving an accuracy of 0.8530 across six segmentation classes in average. This performance is comparable to state-of-the-art results in various segmentation tasks [16] and demonstrates acceptable accuracy for analyzing relative eye features. The mIoU metric algorithm is described in Algorithm of mIoU calculation. We used the publicly available DeepLabV3 + training code [17] from GitHub, which can reproduce the segmentation model with similar datasets.

Algorithm of mIoU calculation (Pseudo-code)

1. Initialize total_intersection and total_union as zero arrays of length C (number of classes)
2. for each image i in dataset do
3. for each class c in C do
4. GTc ←  ground truth mask for class c
5. Predc ←  predicted mask for class c
6. intersectionc ←  | GTc ∩  Predc | 
7. unionc ←  | GTc ∪  Predc | 
8. total_intersection[c] += intersection
9. total_union[c] += unionc
10. end for
11. end for
12. for each class c in C do
13. IoUc ←  total_intersection[c]/total_union[c]
14. end for
15. mIoU ←  (1/C) Σc = 1 to C IoUc
16. return mIoU

#### Step 2: Circle detection and masking.

To accurately measure circular features like the iris and pupil, we applied a well-established circle detection algorithms [18,19]. This method identifies the center coordinates (x, y) and radius of circular objects, such as the pupil and iris, through the following process:

1. Edge Detection: An edge detection algorithm, Canny [20] is applied to the image, emphasizing the contours of circular structures such as the iris. Canny edge detection is a multi-step process to detect edges in an image. It involves gaussian filter to smoothing the image, finding intensity gradients, applying non-maximum suppression to thin edges, and using double thresholding and edge tracking to finalize edges. This ensures precise and continuous edge detection. See Algorithm of mIoU calculation for the pseudo-code of this process.

Algorithm of Canny Edge Detection (Pseudo-code).

1: Input: Grayscale image
2: Smooth image with Gaussian filter
3: Compute intensity gradients:
4: Gx ←  Sobel operator in x direction
5: Gy ←  Sobel operator in y direction
6: G ←  √ (Gx² +  Gy²)
7: θ ←  atan2(Gy, Gx)
8: Apply Non-Maximum Suppression
9: for each pixel p do
10: Compare G[p] with neighbors along θ[p]
11: if G[p] is not local maximum then
12: G[p] ←  0
13: end if
14: end for
15: Apply Double Thresholding:
16: Define Thigh and Tlow
17: for each pixel p do
18: if G[p] >  Thigh then
19: mark p as strong edge
20: else if G[p] >  Tlow then
21: mark p as weak edge
22: else
23: G[p] ←  0
24: end if
25: end for
26: Edge Tracking by Hysteresis:
27: for each weak edge pixel w do
2. Voting in Hough Space: For each detected edge pixel (x, y), the algorithm calculates possible circles by voting in the Hough parameter space. This process involves varying the radius (r) and identifying the center points (a, b) using the equations: a =  x − rcos(θ), b =  y − rsin(θ) where θ ranges from 0° to 360°.
3. Circle Identification: Peaks in the Hough parameter space are detected, corresponding to the most likely circle centers and radii.
4. Return Values: The algorithm returns the coordinates (x, y) and radius of the detected circles.

After circling process, software generates segmentation masks. Several masks were created for the EER calculation.

1. Cornea mask: Created by combining the pupil and iris masks.
2. Exposed eye mask: Formed by merging the cornea mask with the white area mask.
3. Full iris mask: A boolean mask initialized based on the iris radius and center coordinates.
4. Exposure eye area mask: Combined exposed area of cornea, sclera and white area
5. Whole area mask: Combined the white area, sclera, cornea, and adjusted eyelid curve. The eyelid curve is drawn as a natural arc using a Bézier curve that smoothly encompasses the protrusion of the full iris and white area.

#### Step 3: Cornea Exposure Ration (CER) calculation.

To compute the Corneal Exposure Ratio (CER), we first detected the pupil and iris using the circle detection method. We then created a cornea mask by combining the detected pupil and iris with their respective masks. The white area of the eye was also segmented to further refine the exposed eye mask.

Using a Boolean mask, we initialized the full iris mask by setting each pixel within the iris radius to True. The cornea mask was then updated by performing a logical AND operation between the full iris mask and the exposed eye mask.

The final step involved calculating the CER as the ratio of the cornea area to the full iris area. This was achieved by counting the non-zero elements in the respective masks and multiplying the result by 100 to obtain the percentage of exposed cornea.


$$CER=\frac{CorneaMask}{FullIrisMask}\left(100\right)$$ (1)

#### Step 4: Medial and Lateral White Area Ratios (MWR & LWR).

To calculate the Medial White Area Ratio (MWR) and Lateral White Area Ratio (LWR), the white areas on either side of the pupil were measured using the segmented white area mask. For the MWR, we measured the white area to the left (for left eyes) or right (for right eyes) of the pupil center. Similarly, for the LWR, the white area on the opposite side of the pupil was measured. The total white area was obtained by adding the non-zero elements in the white area mask and cornea mask. The formulas for MWR and LWR were as follows:


$$MWR=\frac{MedialWhiteAreaMask}{TotalWhieAreaMask}\left(100\right)$$ (2)

$$LWR=\frac{LateralWhiteAreaMask}{TotalWhieAreaMask}\left(100\right)$$ (3)

#### Step 5: Eyeball Exposure Area (EEA) and Eyeball Exposure Rate (EER).

The Eyeball Exposure Area (EEA) was determined by segmenting the sclera and corneal regions from the entire eye area. EER is calculated by taking the ratio of the segmented eye area to the total area, providing a percentage-based measure of exposure.


$$EER=\frac{EEAMask}{WholeAreaMask}\left(100\right)$$ (4)

### Statistical analyses

All data were analyzed using the Statistical Package for the Social Sciences (SPSS) version 26 (IBM Corporation, Armonk, NY, USA) and Microsoft Excel version 16.84 (Microsoft®, Redmond, WA, USA). Statistical significance was defined as p <  0.05.

The mean values of EER measurements were calculated, and the differences between pre- and post-surgery EER values were analyzed using a Two-Paired t-test. The reliability between the AI-tool and manual measurements of EER was analyzed using a one-way ANOVA test. Intraclass correlation coefficients (ICCs) were used to quantify the agreement between CER measurements obtained from ImageJ and the AI-tool, as well as between the AI tool’s first and second EER measurements. The ICC values were interpreted as follows: values between 0.41 and 0.6 indicated moderate agreement, values between 0.6 and 0.8 indicated substantial agreement, and values between 0.8 and 1.0 indicated excellent agreement. Bland-Altman plots were generated to visually compare the differences between EER measurements obtained from ImageJ and the AI-tool, as well as between the first and second EER measurements performed by the AI-tool. The improvement rate of CER before and after surgery based on ptosis severity was analyzed using a two-way ANOVA test. The complete dataset containing the preoperative and postoperative EER values for all study participants is available in S1 File.xlsx (Supporting Information).

## Results

### Pre- and post-operative EER comparison

**Fig 4:**
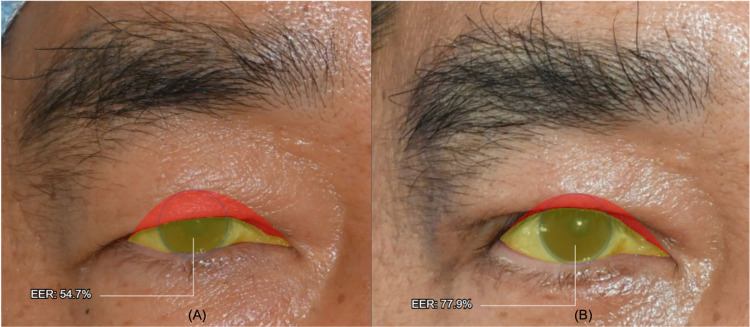
EER values before and after surgery images from the same patient using AI-tool (Anigma-View 1.0.6). (A) Before surgery EER value generated by the AI-tool, (B) After surgery EER value generated by the AI-tool.

The Eyeball Exposure Rate (EER) values (mean ±  standard deviation) measured using both the repeated AI-tool (Fig 4) measurement and Manual (ImageJ) measurement for 100 eyes, pre- and post-surgery, are summarized in Table 2. Before surgery, the repeated AI-tool measurement of EER was 58.85% ±  9.74%, while the Manual (ImageJ) measurement yielded a similar value of 58.22% ±  9.79%. After surgery, the EER measured by the repeated AI-tool was 75.36% ±  7.75%, and the Manual (ImageJ) measurement recorded a value of 75.27% ±  7.93%. Significant differences between the pre- and post-operative EER values were observed across all measurement methods (AI-tool 1st, AI-tool 2nd, and Manual (ImageJ)) as determined by the paired t-test (p <  .001). These results indicate improvement in EER following ptosis surgery.

**Table 2:** Pre-operation and Post-operation repeated AI-based and Manual (ImageJ) measurements of EER.

	Pre-operation	Post-operation	t
**AI-tool 1st**	58.85 ± 9.74	75.36 ± 7.75	−16.653***
**AI-tool 2nd**	58.87 ± 9.74	75.36 ± 7.75	−16.640***
**Manual (ImageJ)**	58.22 ± 9.79	75.27 ± 7.93	−17.070***

Statistical significances were evaluated by Two-Paired t-test, Mean ±  Standard Deviation.

*: p <  .05,

**: p <  .01;

***: p <  .001.

### Agreement between AI-based and manual (ImageJ) measurements of EER

**Fig 5:**
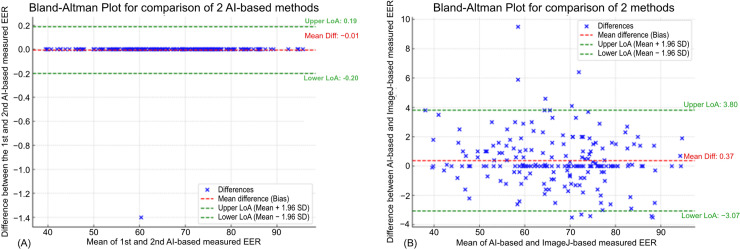
Bland-Altman analysis between different EER measurements. (A) Bland-Altman plot for AI-tool 1st and 2nd measurement, (B) Bland-Altman plot for AI-tool and Manual (ImageJ) measurement.

To assess the agreement between AI-based and Manual (ImageJ) measurements of EER, three measurements were taken using both methods. The EER of a total of 100 eyes was measured during each of the three measurements. The results demonstrated no significant differences between the three measurements, as determined by a one-way ANOVA test (p >  0.5) (Table 3). The Intraclass Correlation Coefficients (ICCs) between Manual (ImageJ) and AI-based measurements in pre- and post-operation ranged from 0.984 to 0.994 and from 0.984 to 0.993, respectively, with all p-values <  .001 (Table 4). This indicates excellent agreement between the two methods. Additionally, the ICCs between the two repeated AI-based measurements reached 1 (p <  .001), reflecting the AI-tool’s high repeatability. The Bland-Altman plot (Fig 5) also confirmed the AI-tool’s high reproducibility, with a bias range of − 0.20 to 0.19 between the two repeated AI-based measurements. Similarly, the bias range between the manual (ImageJ) and AI-based measurements of EER ranged from − 3.07 to 3.80, indicating excellent agreement between the two methods.

**Table 3:** The comparison of EER values based according to measurement method, with Pre- and Postoperative Averages for Total Eyes (n = 100).

	EER values	F
**AI-tool 1st**	67.11 ± 12.06	.061
**AI-tool 2nd**	67.11 ± 12.06	.061
**Manual (ImageJ)**	66.74 ± 12.33	.061

Statistical significances were evaluated by One-Way ANOVA, Mean ±  Standard Deviation.

*: p <  .05,

**: p <  .01;

***: p <  .001.

**Table 4:** Intraclass correlation coefficients(ICC) between two measurements of EER pre-operation and post-operation.

		ICC	(95% CI)
**Pre-operation**			
	**AI-1st & AI-2nd**	1.000	(1.000–1.000)***
	**AI-1st & Manual (ImageJ)**	.990	(.984–.994)***
**Post-operation**			
	**AI-1st & AI-2nd**	1.000	–
	**AI-1st & Manual (ImageJ)**	.990	(.984–.993)***

*: p <  .05,

**: p <  .01;

***: p <  .001.

–: When the data of the two variables match.

### EER improvement rate by ptosis severity

**Fig 6:**
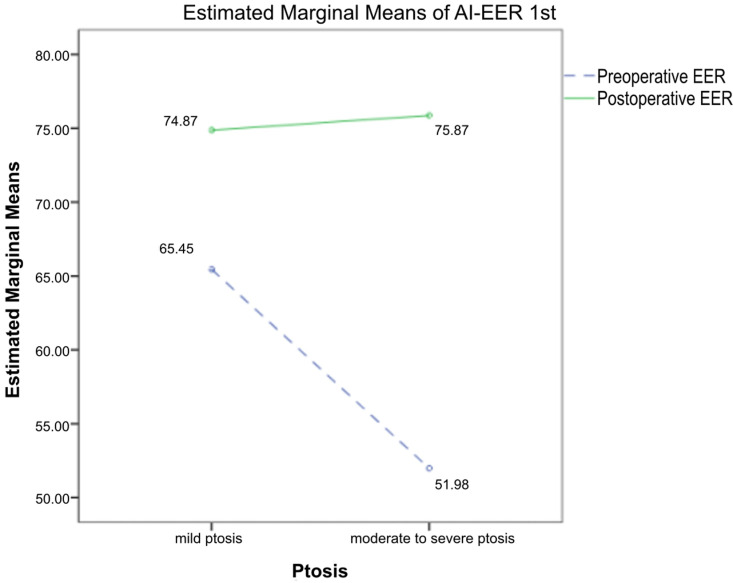
The comparison of EER values pre- and post-operation according to ptosis severity using 1st AI-tool.

Changes in EER values pre- and post-surgery based on ptosis severity measured by 1st AI-tool are summarized in Table 5. Among the 100 eyes analyzed from 50 patients, 51 eyes were classified as mild and 49 eyes as moderate to severe. In the mild ptosis group, the EER increased by 14.39%, from 65.45% ±  6.96% to 74.87% ±  8.06%. In the moderate to severe group, EER increased from 51.98% ±  7.11% to 75.87% ±  7.47%, representing a 45.94% improvement (Fig 6). These findings indicate significant EER improvements in both groups after surgery (p <  .001), with a greater improvement rate observed in the moderate to severe group compared to the mild ptosis group.

**Table 5:** The comparison of EER values pre- and post-operation according to ptosis severity using 1st AI-tool.

	Pre-operation	Post-operation	F
**Total eyes (N = 100)**	58.85 ± 9.74	75.36 ± 7.75	35.368***
**Mild (N = 51)**	65.45 ± 6.96	74.87 ± 8.06	35.368***
**Moderate to Severe (N = 49)**	51.98 ± 7.11	75.87 ± 7.47	35.368***

Statistical significances were evaluated by Two-Way ANOVA, Mean ±  Standard Deviation.

*: p <  .05,

**: p <  .01;

***: p <  .001.

## Discussion

In plastic surgery, particularly in procedures involving the eyes, such as ptosis surgery, the accurate evaluation of surgical outcomes is crucial. Accurate assessment before and after surgery is essential for both improving surgical outcomes and enhancing patient satisfaction. Precise pre- and post-operation assessments allow surgeons to objectively measure the improvements brought about by the surgery, enabling them to make informed decisions about the efficacy of the procedure. Furthermore, patients gain reliable evidence of improvement, which can lead to increased satisfaction with the procedure.

Previous studies have highlighted the utility of EER as an essential eyelid metric and the effectiveness of AI-based methods in evaluating eyelid parameters. Menglin Lu et al. [2] quantified changes in the EER as an important metric for evaluating blepharoplasty outcomes, revealing significant increases in EER. These findings underscore this metric’s ability to objectively evaluate surgical results. Ki Soo Park et al. [4] expanded on this by establishing normal EEA values across different age and gender groups in a Korean cohort, emphasizing the influence of demographic factors on eyelid morphology and providing a baseline for evaluating surgical interventions. More recently, AI-based approaches have gained attention. Yoonsoo Nam et al. [7] introduced a Neural-network technology for measuring Margin Reflex Distances (MRD1 and MRD2), achieving high reliability (ICC >  0.9) and demonstrating the advantages of automation in reducing interobserver variability and processing time. Similarly, Ji Shao et al. [6] utilized Deep-learning to assess eyelid morphology, specifically analyzing Midpupil Lid Distances (MPLD), with results that showed strong agreement with manual methods. These advancements highlight AI’s potential for precise and scalable analysis. Such studies provide critical context for the present research, which aims to integrate and evaluate these methods for pre- and post-operative EER measurement in ptosis surgery, with a focus on clinical reliability and demographic applicability.

This study is significant in comparing the conventional ImageJ method with an AI-tool for measuring EER before and after ptosis surgery. The findings demonstrate that AI- tools yield EER measurements comparable in accuracy to those obtained using ImageJ, with no significant differences between the two methods. While AI-tool matches the performance of conventional methods, it offers distinct advantages, particularly in terms of speed and efficiency.

Metrics such as Marginal Reflex Distance (MRD) and Palpebral Fissure Height (PFH) have been used in ptosis surgery evaluations. However, these metrics are one-dimensional and may not fully capture the three-dimensional changes in the eye’s appearance that often result from surgery. Additionally, newer eye surgeries, such as medial epicanthoplasty and lateral canthoplasty, focus on increasing the eyeball area, resulting in a more three-dimensional and enlarged eye appearance [3]. Nowadays, these surgeries are becoming more common because they make the eyes look bigger and more attractive [21]. These surgeries need more comprehensive metrics, as conventional MRD and PFH are inadequate to reflect these changes. When measuring MRD1 manually using ImageJ, there may be difficult situations, especially in cases where the pupil and iris are not clearly distinguishable, such as in individuals with brown iris [7]. This problem may lead to inaccuracies when determining the pupil center, which affects the measurement’s overall accuracy. In comparison, the Corneal Exposure Area (CER) is a two-dimensional metric and does not rely on the vertical distance passing through the iris. However, there is a limitation in its ability to measure the full ocular surface, including the scleral area [4]. In this respect, the EER offers significant usefulness over MRD, PFH and CER. By not relying on the pupil center, EER is more robust and avoids some of the issues associated with MRD measurement, especially in patients with dark iris [3,4]. In addition, it includes the scleral area, providing a more comprehensive two-dimensional measurement that better reflects three-dimensional changes in the eye’s size and shape.

While ImageJ has been widely used as a tool for objective image analysis, its reliance on manual measurement introduces variability based on the skill and experience of the evaluator [7]. This manual process is also labor-intensive and time-consuming, making it less ideal for real-time clinical use where speed and efficiency are important. AI-tools, on the other hand, offer significant improvements over conventional methods. AI systems can automate image analysis, providing faster, more consistent results without relying on the evaluator’s expertise and manual intervention [6–9]. AI-tools are particularly useful in research and clinical settings where large datasets of images need to be processed efficiently. The ability to process images quickly and accurately makes AI-tools a promising solution for both research and clinical applications.

Despite these advantages, the adoption of AI in medical image analysis also presents significant challenges. A primary challenge is the dependency on high-quality training datasets [7], which are often insufficient or lack diversity, potentially compromising the generalizability of AI systems across different populations. Furthermore, the interpretability of deep learning models remains a critical concern, as these systems often operate as non-transparent systems, making it difficult for clinicians to comprehend their decision-making processes. Such opacity can erode trust in AI tools, especially in high-stakes clinical applications [9]. Additionally, biases inherent in training data can propagate through AI systems, resulting in inconsistent performance and inequitable outcomes across demographic groups [9]. Thus, while AI has the potential to revolutionize medical image analysis, these challenges highlight the importance of rigorous validation and transparency to ensure its effectiveness and equity in diverse clinical settings.

This study does have several limitations that should be considered. First, the sample size was relatively small, and the study only included Korean patients with ptosis, which may affect the generalizability of the findings. Similarly, the DeepLabV3 + model was trained on a limited dataset of only 396 eye images, and despite implementing data augmentation techniques, the model might show limited performance when applied to significantly different visual characteristics, particularly across different ethnicities and age groups. Therefore, further study is needed to explore a larger number of patients of different ethnicities and different medical conditions. Second, the study was conducted at a single center, which could introduce biases related to specific practices or patient populations. Finally, the AI-tools used in this study analyzed only two-dimensional images, limiting their ability to fully capture the three-dimensional changes in eye appearance following surgery. So, considering the stereoscopic changes in the EER of the eye, two-dimensional analysis potentially affects the accuracy of the measurements.

Despite these limitations, AI-tools hold enormous promise for the future of surgical assessment. They could be used to measure an expanded range of surgical metrics, such as MRD and PFH, and could also be applied to other types of surgeries, including medial and lateral canthoplasty, which affect both the horizontal and vertical dimensions of the eye. The methodology developed in this study can be easily extended to a broader range of surgical assessments beyond eyelid surgery.

## Conclusion

In conclusion, AI-based methods provide a faster, more efficient, and similarly accurate to conventional manual methods like ImageJ for measuring EER. These tools offer significant advantages in both clinical and research settings, allowing for more objective evaluations, processing large volumes of images quickly and providing consistent measurements. And these advantages can enable researchers to perform more precise and impactful studies in the field of plastic and reconstructive surgery. Moreover, AI-based methods have the potential to increase the objectivity of surgical outcomes, thereby improving both patient satisfaction and the surgeon’s ability to produce precise results. Future studies need to explore the potential of AI-based methods to analyze three-dimensional changes in the eye to further improve the accuracy of surgical assessments. Additionally, the use of AI-tools across various types of cosmetic and reconstructive surgeries could enhance both the speed and precision of postoperative evaluations.

## Supporting information

S1 File The dataset of EER values. The preoperative and postoperative EER values for all study participants, obtained from both the Manual method (ImageJ) and the AI-tool.
(XLSX)
